# Mechanisms of linezolid resistance in staphylococci and enterococci isolated from two teaching hospitals in Shanghai, China

**DOI:** 10.1186/s12866-014-0292-5

**Published:** 2014-11-25

**Authors:** Yueru Tian, Tianming Li, Yuanjun Zhu, Bei Wang, Xue Zou, Min Li

**Affiliations:** Department of Laboratory Medicine, Huashan Hospital, Shanghai Medical College, Fudan University, 12 Central Urumqi Road, Shanghai, China; Department of Laboratory Medicine, Renji Hospital, Shanghai Jiaotong University School of Medicine, Shanghai, China; Department of Laboratory Medicine, the Second Affiliated Hospital, Jiangxi University of Traditional Chinese Medicine, Nanchang, China

**Keywords:** Linezolid resistance, Mutations of 23S rRNA and ribosomal proteins, cfr, Biofilm production, Cell wall thickness

## Abstract

**Background:**

Linezolid is one of the most effective treatments against Gram-positive pathogens. However, linezolid-resistant/intermediate strains have recently emerged in worldwide. The purpose of this study was to analyse the prevalence and resistance mechanisms of linezolid-resistant/intermediate staphylococci and enterococci in Shanghai, China.

**Results:**

Thirty-two linezolid-resistant/intermediate strains, including 14 *Staphylococcus capitis*, three *Staphylococcus aureus*, 14 *Enterococcus faecalis* and one *Enterococcus faecium* clinical isolates, were collected in this study which displayed linezolid MICs of 8 to 512 μg/ml, 8–32 μg/ml, 4–8 μg/ml and 4 μg/ml, respectively. All linezolid-resistant *S. capitis* isolates had a novel C2131T mutation and a G2603T mutation in the 23S rRNA region, and some had a C316T (Arg106Cys) substitution in protein L4 and/or harboured *cfr*. Linezolid-resistant *S. aureus* isolates carried a C389G (Ala130Gly) substitution in protein L3, and/or harboured *cfr*. The *cfr* gene was flanked by two copies of the IS256-like element, with a downstream *orf1* gene. Linezolid-resistant/intermediate enterococci lacked major resistance mechanisms. The semi-quantitative biofilm assay showed that 14 linezolid-resistant *E. faecalis* isolates produced a larger biofilm than linezolid-susceptible *E. faecalis* strains. Transmission electron microscopy showed the cell walls of linezolid-resistant/intermediate strains were thicker than linezolid-susceptible strains.

**Conclusion:**

Our data indicated that major resistance mechanisms, such as mutations in 23S rRNA and ribosomal proteins L3 and L4, along with *cfr* acquisition, played an important role in linezolid resistance. Secondary resistance mechanisms, such as biofilm formation and cell wall thickness, should also be taken into account.

## Background

Gram-positive cocci pose a worldwide threat to human health. The emergence of antibiotic resistance in Gram-positive cocci, including methicillin-resistant *Staphylococcus aureus* (MRSA), vancomycin-resistant staphylococci (VRS) and vancomycin-resistant enterococci (VRE), has created a clinical demand for effective novel therapeutic agents. Linezolid (LZD), the first member of the oxazolidinone class of antibiotics, was approved for clinical use in 2000 and has a broad spectrum of activity against a variety of Gram-positive pathogens. It acts by inhibiting protein synthesis via binding to the peptidyl transferase centre of the 50S ribosomal subunit, and preventing formation of the fMet-tRNA-30S ribosome-mRNA initiation complex [1]. Because of its unique antimicrobial mechanism, linezolid has been widely applied in the treatment of clinically-important Gram-positive bacteria, including aerobic and anaerobic Gram-positive cocci, aerobic and anaerobic Gram-positive bacilli, and nocardia and mycobacteria species. However, linezolid-resistant (LR) staphylococcus was first reported in peritonitis patients undergoing oral linezolid treatment during peritoneal dialysis in 2001 [2]. Since then, the occurrence of LR strains has been reported worldwide [3-5].

The major mechanism of resistance to linezolid is caused by mutations in the V domain of the 23S rRNA gene, with a G2576T substitution (*Escherichia coli* numbering) occurring most frequently. C2104T, G2447T, T2500A, A2503G, T2504A, G2603T and G2631T substitutions have also been found in LR strains [6-9]. Another resistance mechanism is horizontal acquisition of *cfr*, which encodes a methyltransferase and modifies adenosine at A2503 in the 23S rRNA. *cfr* is usually plasmid-located and confers cross-resistance to phenicol, lincosamide, oxazolidinone, pleuromutilin and streptogramin A (known as the PhLOPSA phenotype) [10,11]. Alterations in the ribosomal proteins L3, L4 and L22, encoded by *rplC*, *rplD* and *rplV*, respectively, have also been associated with increased resistance to linezolid [12-14]. In addition, secondary resistance mechanisms, such as biofilm formation and cell wall thickening, can enhance resistance to antibiotics as well [15,16].

As the global emergence of LR isolates has increased [2-7], LR Gram-positive cocci have also become a problem in China [8,17-19]. To comprehensively understand the current prevalence and resistance mechanisms among LR clinical isolates in China, we analysed 32 linezolid-resistant/intermediate strains, including 14 *Staphylococcus capitis*, three *S. aureus*, 14 *Enterococcus faecalis* and one *Enterococcus faecium* isolates.

## Methods

### Ethics statement

The collection of the linezolid-resistant/intermediate bacterial isolates from patients and the related information of patients were approved by the ethics committee of Huashan Hospital, Shanghai Medical College, Fudan University and the ethics committee of Renji Hospital, Shanghai Jiaotong University School of Medicine, Shanghai, People’s Republic of China. All subjects provided written informed consent before their inclusion in the study.

### Bacterial isolates

Thirty-two non-duplicated linezolid-resistant/intermediate isolates were collected from patients of two Shanghai comprehensive teaching hospitals in China from 2009–2013. One of the hospitals was Huashan Hospital, which is a tertiary care hospital affiliated with Fudan University, located in the centre of Shanghai. It is one of the largest (1300 beds) teaching hospitals in china, handling approximately 8000 admissions per day. The other was Renji Hospital, a tertiary care hospital affiliated with Shanghai Jiaotong University, which is located in the east of Shanghai, and is also one of the largest (1800 beds) teaching hospitals in china, handling about 9000 admissions per day. The studied isolates comprised 14 *S. capitis* (from Huashan Hospital), three *S. aureus* (from Huashan hospital), 14 *E. faecalis* (12 isolates from Huashan Hospital and two from Renji Hospital) and one *E. faecium* (from Huashan Hospital). Among these 32 isolates, 15, 10, 3, 2, 1 and 1 were recovered from patients with bacteraemia, urinary tract infection, pneumonia, wound infection, biliary tract infection and prostate infection, respectively (Table 1). Isolates were identified using a VITEK 2 compact system (bioMérieux, Marcy l’Etoile, France) and a molecular method based on analysis of the 16S rRNA gene sequence. *S. aureus* RN4220 was used as the recipient strain for transformation experiments. *S. aureus* ATCC 29213, *E. faecalis* ATCC 29212 (both with linezolid MIC of 2 μg/ml, American type culture collection, USA), *S. capitis* HS12-102 and *E. faecium* HS13-194 (both with linezolid MIC of <2 μg/ml, from Huashan Hospital) were included as linezolid-susceptible (LS) strains. All strains were stored at −70°C until use and were incubated overnight on blood agar at 37°C.

**Table 1:** **Clinical characteristics of patients with linezolid-resistant** ***Staphylococcus*** **or** ***Enterococcus*** **infection**

**Strains**	**Organism**	**Underlying diseases**	**Infection type**	**Ward**	**Prior antibiotic use**	**Outcome**
HS09-206	*S. capitis*	Haematencephalon,Hypertension, Renal failure	Bacteraemia	Intensive care unit (ICU)	SCF.AMK.MTZ	Discharge
HS09-17	*S. capitis*	Severe craniocerebral injury, Infective shock	Bacteraemia, Pneumonia,	Department of Cerebral Surgery	MEN.LZD.SCF. DOX	Discharge
HS10-204	*S. capitis*	Craniocerebral injury,	Bacteraemia, Pneumonia	ICU	SXT.VAN	Discharge
HS10-24	*S. capitis*	Hip joint surgery,	Bacteraemia, Diffuse peritonitis	ICU	TZP.MEN.LZD. LVX.SCF	Discharge
HS11-203	*S. capitis*	Bladder surgery, Infective shock	Bacteraemia	ICU	MEN.VAN	Discharge
HS11-44	*S. capitis*	Subarachnoid haemorrhage	Bacteraemia	ICU	LZD	Discharge
HS12-49	*S. capitis*	Craniocerebral injury,	Bacteraemia, Pneumonia	ICU	VAN.SCF	Discharge
HS12-201	*S. capitis*	Ventricular fibrillation, Epilepsy	Bacteraemia	ICU	MEN.SCF.AMK. TZP	Discharge
HS12-51	*S. capitis*	Circulatory failure,	Bacteraemia, Pneumonia	ICU	IPM.LZD.VAN. FEP.TEC.CAZ. LVX.AMK	Discharge
HS12-53	*S. capitis*	Craniocerebral injury	Bacteraemia	Department of Cerebral Surgery	MEN.AMK.SAM.VAN	Discharge
HS12-55	*S. capitis*	Acute respiratory failure, Nephrosis syndrome	Bacteraemia, Pneumonia,	ICU	IPM.FOF.MEN. SCF.DOX	Discharge
HS12-58	*S. capitis*	Subarachnoid haemorrhage	Bacteraemia	ICU	CXM.SCF	Discharge
HS13-60	*S. capitis*	Brain tumour operation, Respiratory failure	Bacteraemia	ICU	MEN.LZD.SCF. DOX	Discharge
HS13-207	*S. capitis*	Subarachnoid haemorrhage	Bacteraemia	ICU	IPM.LZD.VAN. FEP.TEC.CAZ. LVX.AMK	Discharge
HS09-205	*S. aureus*	COPD, Coronary disease	Pneumonia	General ward	SCF	Discharge
HS11-202	*S. aureus*	Hematencephalon	Pneumonia	ICU	SCF	Discharge
HS12-56	*S. aureus*	Craniocerebral injury	Pneumonia	ICU	VAN.SCF	Discharge
HS10-311	*E. faecalis*	Urinary tract infection	Urinary infection	Department of Urology (outpatient)	N/day	Infection cleared
HS11-304	*E. faecalis*	Hepatocellular carcinoma after operation	Bacteraemia, Infectious endocarditis	Department of Infectious Disease	VAN.FOF.SAM. AMK.MTZ	Discharge
HS11-305	*E. faecalis*	Urinary tract infection	Urinary infection	Department of Urology (outpatient)	N/D	Infection cleared
HS11-307	*E. faecalis*	Chronic subdural hematoma	Urinary infection, Pneumonia	Department of Cerebral Surgery	MEN.LZD.SCF. DOX	Discharge
HS12-309	*E. faecalis*	Urinary tract infection	Urinary infection	Department of Urology (outpatient)	N/D	Infection cleared
HS12-308	*E. faecalis*	Urinary tract infection	Urinary infection	Department of Urology (outpatient)	N/D	Infection cleared
HS12-310	*E. faecalis*	Femoral fracture	Wound infection	Department of Orthopaedics	MEN	Discharge
HS13-301	*E. faecalis*	Urinary tract infection	Urinary infection	Department of Urology (outpatient)	N/D	Infection cleared
HS13-302	*E. faecalis*	Scrofula	Biliary tract infection	Department of Infectious Disease	MXF.SCF.IHY.PYR.	Discharge
HS13-303	*E. faecalis*	Drug fever	Urinary infection	Department of Infectious Disease	AZM	Discharge
RJ13-314	*E. faecalis*	Juvenile arthritis	Urinary infection	Department of Urology (outpatient)	N/D	Infection cleared
RJ13-315	*E. faecalis*	Chronic prostatitis	Prostate infection	Department of Urology (outpatient)	N/D	Infection cleared
HS13-312	*E. faecalis*	Psoriasis	Wound infection	Department of Dermatology	Not used	Discharge
HS13-313	*E. faecalis*	Urinary tract infection	Urinary infection	Department of Urology (outpatient)	N/D	Infection cleared
HS11-306	*E. faecium*	Lithangiuria	Urinary infection	Department of Urology	TZP.SAM.VAN.LVX.CXM	Discharge

HS, isolate from Huashan Hospital; RJ, isolate from Renji Hospital; COPD, chronic obstructive pulmonary disease; SCF, cefoperazone/sulbactam; AMK, amikacin; MTZ, metronidazole; MEM, meropenem; LZD, linezolid; DOX, doxycycline; SXT, sulfamethoxazole/trimethoprim; VAN, vancomycin; TZP, piperacillin/tazobactam; LVX, levofloxacin; IPM, imipenem; FEP, cefepime; TEC, teicoplanin; CAZ, ceftazidime; SAM, ampicillin/sulbactam; FOF, fosfomycin; CXM, cefuroxime; MXF, moxifloxacin; IHY, isonicotinyl hydrazide; PYR, pyrazinamide; AZM, Azithromycin. N/D, not determined.

### Antibiotic susceptibility testing

The antimicrobial agents tested were linezolid, vancomycin, teicoplanin, oxacillin, cefoxitin, trimethoprim-sulfamethoxazole, erythromycin, clindamycin, chloramphenicol, tetracycline, ciprofloxacin, penicillin, ampicillin and high-level gentamicin. The MIC of each antimicrobial agent was determined by the broth microdilution MIC method and interpretation of MIC results was based on 2013 Clinical and Laboratory Standards Institute guidelines [20]. *S. aureus* ATCC 29213 was tested concurrently for quality control.

### Multilocus sequence typing (MLST) analysis

*S. aureus, E. faecalis* and *E. faecium* were screened using a previously described method [21-23] to detect the seven housekeeping genes. For *S. aureus,* there are carbamate kinase (*arcC*), shikimate dehydrogenase (*aroE*), glycerol kinase (*glp*), guanylate kinase (*gmk*), phosphate acetyltransferase (*pta*), triosephosphate isomerase (*tpi*), and acetyl coenzyme A acetyltransferase (*yqiL*). For *E. faecalis,* there are glucose-6-phosphate dehydrogenase (*gdh*), glyceraldehydes-3-phosphate dehydrogenase (*gyd*), phosphate ATP binding cassette transporter (*pstS*), glucokinase (*gki*), shikimate-5-dehydrogenase (*aroE*), xanthine phosphoribosyltransferase (*xpt*), and acetyl-CoA acetyltransferase (*yiqL*). For *E. faecium,* there are adenylate kinase (*adk*), ATP synthase, alpha subunit (*atpA*), d-alanine:d-alanine ligase (*ddl*), glyceraldehyde-3-phosphate dehydrogenase (*gyd*), glucose-6-phosphate dehydrogenase (*gdh*), phosphoribosylaminoimidazol carboxylase ATPase subunit (*purK*), and phosphate ATP-binding cassette transporter (*pstS*). Each sequence was submitted to the MLST website (http://www.mlst.net) to determine the allelic profile and sequence type (ST) of each isolate [24].

### PFGE

PFGE was performed according to a previously described method [25] with some modifications. The staphylococci were treated with lysostaphin (Sigma-Aldrich, Saint Louis, MO, USA) and genomic DNA was prepared in agarose blocks and then digested with the restriction enzyme *Sma*I (NEB, Hitchin, UK). The DNA fragments were separated using a CHEF-DR II PFGE system (Bio-Rad, Hercules, CA, USA) for 20 h at 6 V/cm and 14°C, with a pulse angle of 120° and pulse times from 5–25 s. PFGE banding patterns were analysed visually.

### Molecular detection of resistance genes and mutations

Isolates were screened for the presence of *cfr* and mutations in the 23S rRNA and the L3, L4 and L22 ribosomal proteins by PCR and DNA sequencing, as previously described [26,27]. Amplicons were sequenced on both strands and were compared with those from *S. aureus* ATCC 29213, *E. faecalis* ATCC 29212, LS *S. capitis* and LS *E. faecium*, obtained from Shanghai Huashan Hospital during the study period, using the Lasergene software package (DNAStar; Madison, WI, USA).

### Gene dosage

Gene dosage was determined according to a previously described method [28,29] with some modifications. Isolates of LR *S. capitis* containing mutations in the central loop of the 23S rRNA gene were amplified using primers based on the *S. capitis* 23S rRNA gene (primer F, 5′-AAGGCGTAACGATTTGGG-3′; primer R, 5′-CAGCACTTATCCCGTCCA-3′; expected PCR product size: 720 bp). Thermal cycler conditions were 94°C for 5 min, followed by 35 cycles of 94°C (30 s), 55°C (30 s) and 72°C (30 s), with a final extension at 72°C (7 min). The DNA concentration of PCR amplicons was normalized to 0.3 pmol prior to cloning. Then, the amplification products were ligated to plasmid pMD-18 T (Takara Biotechnology, Dalian, China) and transformed into *E. coli* DH5α cells (Sangon Biotech, Shanghai, China). Each LR *S. capitis* was cloned and 30 clones from each strain were sequenced.

### Plasmid analysis and cfr location

Plasmid DNA was extracted from all *cfr*-carrying isolates using a Plasmid DNA Midi Kit (QIAGEN, Hilden, Germany) and separated on 1% (w/v) agarose gel. The migration distances of DNA bands were measured directly from gel photographs in relation to the reference plasmids in E. coli V517 (sizes, 54, 5.6, 5.1, 3.9, 3.0, 2.7, and 2.1 kb) marker. Plasmid DNA bands were transferred onto a nylon membrane by Southern blotting. A *cfr*-specific labelled probe (primer F, 5′-GAAGCTCTAGCCAACCGTCA-3′; primer R, 5′-TCTACCTGCCCTTCGTTTGC3′; expected PCR product size: ~600 bp) was generated using a non-radioactive DIG High Prime DNA Labelling and Detection Kit (Roche Applied Sciences, Mannheim, Germany) according to the manufacturer’s instructions, and was hybridised to the membrane. To determine the genetic environment of *cfr*, the partial plasmid DNA sequence of all *cfr*-carrying isolates was determined using the following primers, designed based on the sequence of pSS-01 (GenBank accession no. JQ041372): 1 F 5′- CACTTCCTTTATTATTTTTC-3′; 1R 5′- CGACTGAATCAAGAAGTACG-3′; 2 F 5′-TGAACCATAACCTTTGTC-3′, 2R 5′-TGTTTCTAGCCTGACTGA-3′; 3 F 5′-ACTCGATGGTCCTCACGG-3′, 3R 5′-TCAGGCTCATTATTACTTC-3′; 4 F 5′-TTACCACTAGAGCAAATT′, 4R 5′-GACCACAAGCAGCGYCAA-3′; 5 F 5′-AATGGACTACTGGGTGGA-3′, 5R 5′-ATGATTCAGAAAGCAGAT-3′; 6 F 5′-CTTTCCGAATGGACTAAT-3′, 6R 5′-TGAGCGCAGCTTTACGAC-3′ (PCR product sizes: 1000 ~ 2500 bp). Amplification products from the ten *cfr*-carrying plasmids were sequenced on both strands and were compared with plasmid pSS-01 using the Lasergene software package (DNAStar; Madison, WI, USA). In addition, the purified plasmids extracted from each of the ten original strains were electrotransformed (2kv, 25 μF, 1000Ω) into the *S. aureus* recipient strain RN4220. Transformants were incubated in fresh tryptic soy broth (TSB; Oxoid, Basingstoke, UK) at 37°C with shaking at 200 rpm for 1 h and then were selected by incubation for 24 h at 37°C on TSB agar supplemented with 6 μg/ml linezolid. The linezolid MICs of transformants were measured by the broth microdilution method. Plasmid DNA was extracted from all the transformants and separated on 1% agarose gel. Southern hybridisation was then used to confirm whether the plasmids were *cfr*-positive as described previously [17].

### Detection of biofilm production

All LR strains were divided into four groups by species (*S. capitis*, *S. aureus*, *E. faecalis* and *E. faecium*). *S. capitis* group contained 14 LR isolates and 14 LS isolates. *S. aureus* group contained 3 LR isolates and 15 LS isolates. *E. faecalis* group contained 14 LR isolates and 14 LS isolates. *E. faecium* group contained 1 LR isolate and 15 LS isolates. A semi-quantitative biofilm assay was conducted using flat-bottomed, tissue culture-treated, 96-well assay plates (Costar 3599; California, USA). Four wells in each assay plate contained untreated cultures, which served as negative controls. Bacteria were prepared by making a 1:100 dilution of an overnight culture grown in TSB containing 5 g/L glucose. Assay plates were inoculated with 200 μL of culture per well (OD_600_ = 0.01) and were incubated at 37°C for 24 h. The medium were removed from assay plates. Then assay plates were washed four times with PBS. Biofilms were fixed by Bouin’s fixation for at least 1 h then assay plates were washed four times with PBS. Following crystal violet staining, the biofilms were measured using a Synergy H1 Hybrid Multi-Mode Microplate Reader (Biotek, Vermont, USA) at OD_570_.

### Transmission electron microscopy (TEM)

Isolates for TEM were grown from a single colony in 10 mL of liquid brain-heart infusion (BHI) medium with 0.5% (w/v) beef extract. Four groups were studied. The *S. capitis* group contained strains HS12-102 (LS *S. capitis* strain HS12-55), HS09-17 (MIC 16 μg/ml), HS09-17 + LZD (strain HS09-17 grown in BHI broth containing 4 μg/ml linezolid), HS12-55 (MIC >256 μg/ml) and HS12-55 + LZD (strain HS12-55 grown in BHI broth containing 32 μg/ml linezolid). The *S. aureus* group contained *S. aureus* ATCC29213 (LS *S. aureus*), HS11-202 (MIC 16 μg/ml) and HS11-202 + LZD (strain HS11-202 grown in BHI broth containing 2 μg/ml linezolid). The *E. faecalis* group contained *E. faecalis* ATCC29212 (LS *E. faecalis*), HS11-304 (MIC 4 μg/ml), HS12-309 (MIC 8 μg/ml) and HS12-309 + LZD (strain HS12-309 grown in BHI broth containing 2 μg/ml linezolid). Finally, the *E. faecium* group contained strains HS13-194 (LS *E. faecium*) and HS11-306 (MIC 4 μg/ml). The cells were grown at 37°C with shaking at 200 rpm for approximately 4–5 h to mid-exponential phase (OD600 ~ 0.7–0.8). An 8-mL volume of each culture was pelleted by centrifugation at 4000 g for 15 min and suspended in 1 mL of 2.5% (v/v) glutaraldehyde in 0.1 M phosphate buffer, and stored at 4°C for at least 2 h. The cells were then post-fixed with 1% (w/v) osmium tetroxide in the same buffer. Following dehydration, cells were infiltrated with and embedded in Eponate 12 resin for 24 h. Ultrathin sections were cut at 70 nm, stained with uranyl acetate and lead citrate, and then examined on a JEM-1230 80 kV TEM (JEOL, Tokyo, JAPAN) equipped with a Gatan Orius SC200W camera. Images were captured at 30,000× magnification and measurements were made using ImageJ software [30]. Thirty cell walls were measured per sample, and means and standard deviations were calculated in Excel (Microsoft, San Francisco, USA).

## Results

### Incidence of linezolid resistance

From 2009–2013, 3446 *Staphylococcus* and 1713 *Enterococcus* non-duplicated isolates were tested for susceptibility to linezolid. The incidence of linezolid resistance in our study was 1.18% of coagulase-negative staphylococci (CONS) (14/1188 isolates), 0.13% of *S. aureus* (3/2258 isolates), 1.72% of *E. faecalis* (14/812 isolates) and 0.11% of *E. faecium* (1/877 isolates) isolates.

### Antimicrobial susceptibility testing

The susceptibility profiles of 14 *S. capitis* isolates, three *S. aureus* isolates and 14 *E. faecalis* isolates were similar (Table 2). The *S. capitis* isolates showed various levels of linezolid resistance, with MIC values of 8–512 μg/ml. The *S. aureus* isolates were resistant to linezolid, with MIC values of 8–32 μg/ml. Other than *S. aureus* HS09-205, all staphylococcal strains were methicillin-resistant and exhibited high-level resistance to most of the antibiotics tested, except for vancomycin, teicoplanin and tetracycline. Moreover, 17 staphylococcal isolates showed different levels of trimethoprim-sulfamethoxazole susceptibility data, with MIC values of 0.12/2.37–8/152 μg/ml. The fifteen enterococcal isolates demonstrated either linezolid-intermediate (LI) or low-level linezolid resistance (MICs 4–8 μg/ml). These strains were resistant to erythromycin, chloramphenicol, ciprofloxacin and tetracycline (isolate HS11-307 was susceptible to tetracycline, with a MIC of 1 μg/ml), but susceptible to vancomycin (MICs, 0.5–2 μg/ml), teicoplanin (MICs, 0.12–0.25 μg/ml), penicillin (MICs, 0.5–4 μg/ml) and ampicillin (MICs, <0.5–4 μg/ml). Eight enterococcal isolates (HS11-304, HS12-309, HS12-308, HS12-310, HS13-301, HS13-303, RJ13-314 and HS11-306) were not high-level resistant to gentamicin, whereas the remaining strains were resistant.

**Table 2:** **Antimicrobial susceptibility profiles and genetic resistance markers of linezolid-resistant** ***Staphylococcus*** **and** ***Enterococcus*** **isolates**

**Strain ID**	**Source**	**ST**	**MIC (** **μg/ml)**														**Genetic resistance markers**				
			**LZD**	**VAN**	**TEC**	**OXA**	**FOX**	**ERY**	**CLI**	**CHL**	**TET**	**CIP**	**PEN**	**AMP**	**GNH**	**SXT**	**23S rRNA mutation (percentage of clones with mutation)**	***cfr***	**L3**	**L4**	**L22**
*S. capitis*																					
HS09-206	blood	N/D	8	1	0.25	>256	R	>256	>256	32	4	64	64	N/D	N/D	2/38	C2131T (43.3%)	-	-	-	-
																	G2603T (70.0%)				
HS09-17	blood	N/D	16	1	0.25	256	R	>256	256	32	4	64	32	N/D	N/D	2/38	C2131T (46.7%)	-	-	-	-
																	G2603T (83.3%)				
HS10-204	blood	N/D	32	1	0.5	>256	R	>256	>256	128	4	64	64	N/D	N/D	4/76	C2131T (36.7%)	-	-	-	-
																	G2603T (66.7%)				
HS10-24	blood	N/D	64	1	0.25	256	R	>256	>256	32	4	64	32	N/D	N/D	8/152	C2131T (53.3%)	-	-	-	-
																	G2603T (80.0%)				
HS11-203	blood	N/D	256	1	0.25	>256	R	>256	>256	128	4	64	64	N/D	N/D	2/38	C2131T (60.0%)	+	-	-	-
																	G2603T (80.0%)				
HS11-44	blood	N/D	256	1	0.25	>256	R	>256	256	64	4	64	32	N/D	N/D	2/38	C2131T (53.3%)	+	-	-	-
																	G2603T (80.0%)				
HS12-49	blood	N/D	256	1	0.5	>256	R	>256	>256	128	4	64	32	N/D	N/D	4/76	C2131T (70.0%)	+	-	-	-
																	G2603T (70.0%)				
HS12-201	blood	N/D	512	1	0.25	>256	R	>256	>256	128	4	64	64	N/D	N/D	4/76	C2131T (60.0%)	+	-	C316T(Arg106 Cys)	-
																	G2603T (100%)				
HS12-51	blood	N/D	128	1	0.5	256	R	>256	256	32	4	64	64	N/D	N/D	2/38	C2131T (36.7%)	+	-	-	-
																	G2603T (63.3%)				
HS12-53	blood	N/D	512	1	0.25	>256	R	>256	256	128	4	64	32	N/D	N/D	8/152	C2131T (60.0%)	+	-	-	-
																	G2603T (80.0%)				
HS12-55	blood	N/D	512	1	0.25	>256	R	>256	>256	128	4	64	32	N/D	N/D	4/76	C2131T (50.0%)	+	-	C316T(Arg106 Cys)	-
																	G2603T (86.7%)				
HS12-58	blood	N/D	512	1	0.25	>256	R	>256	>256	64	2	32	8	N/D	N/D	4/76	C2131T (60.0%) G2603T (100%)	+	-	-	-
HS13-60	blood	N/D	256	1	0.5	>256	R	>256	>256	64	4	64	64	N/D	N/D	4/76	C2131T (60.0%)	+	-	-	-
																	G2603T (76.7%)				
HS13-207	blood	N/D	32	1	0.25	>256	R	>256	>256	32	4	64	32	N/D	N/D	8/152	C2131T (83.3%)	-	-	-	-
																	G2603T (100%)				
*S. aureus*																					
HS09-205	sputum	88	8	1	0.5	<0.25	S	>256	>256	32	0.5	64	32	N/D	N/D	0.12/2.37	-	-	-	-	-
HS11-202	sputum	239	16	1	1	>256	R	>256	>256	32	4	128	128	N/D	N/D	0.25/4.75	-	-	C389G(Ala130Gly)	-	-
HS12-56	sputum	5	32	1	1	>256	R	>256	>256	32	8	128	8	N/D	N/D	0.12/2.37	-	+	-	-	-
*E. faecalis*																					
HS10-311	urine	16	4	1	0.12	N/D	N/D	>256	N/D	32	128	64	2	<0.5	R	N/D	-	-	-	-	-
HS11-304	blood	16	4	1	0.5	N/D	N/D	>256	N/D	32	64	16	2	<0.5	S	N/D	-	-	-	-	-
HS11-305	urine	476	4	1	0.25	N/D	N/D	>256	N/D	32	128	16	4	<0.5	R	N/D	-	-	-	-	-
HS11-307	urine	4	4	2	0.12	N/D	N/D	>256	N/D	32	1	64	0.5	<0.5	R	N/D	-	-	-	-	-
HS12-309	urine	69	8	2	0.12	N/D	N/D	>256	N/D	32	64	64	0.5	<0.5	S	N/D	-	-	-	-	-
HS12-308	urine	480	8	1	0.25	N/D	N/D	>256	N/D	32	32	64	4	<0.5	S	N/D	-	-	-	-	-
HS12-310	wound	300	8	1	0.25	N/D	N/D	>256	N/D	32	64	64	2	4	S	N/D	-	-	-	-	-
HS13-301	urine	476	8	1	0.25	N/D	N/D	>256	N/D	64	128	64	2	<0.5	S	N/D	-	-	-	-	-
HS13-302	bile	27	8	0.5	0.125	N/D	N/D	>256	N/D	32	128	64	2	<0.5	R	N/D	-	-	-	-	-
HS13-303	urine	480	8	1	0.125	N/D	N/D	>256	N/D	32	64	16	0.5	<0.5	S	N/D	-	-	-	-	-
RJ13 -314	urine	69	8	1	0.12	N/D	N/D	>256	N/D	32	64	16	1	<0.5	S	N/D	-	-	-	-	-
RJ13 -315	urine	4	8	1	0.25	N/D	N/D	>256	N/D	32	128	64	2	<0.5	R	N/D	-	-	-	-	-
HS13-312	wound	37	4	1	0.25	N/D	N/D	>256	N/D	32	64	16	4	4	R	N/D	-	-	-	-	-
HS13-313	urine	480	4	1	0.25	N/D	N/D	>256	N/D	64	128	64	2	<0.5	R	N/D	-	-	-	-	-
*E. faecium*																					
HS11-306	urine	650	4	2	0.25	N/D	N/D	>256	N/D	32	128	16	4	2	S	N/D	-	-	-	-	-

ST, sequence typing; R, resistant; S, susceptible; LZD, linezolid; VAN, vancomycin; OXA, oxacillin; FOX, cefoxitin; ERY, erythromycin; CLI, clindamycin; CHL, chloramphenicol; TET, tetracycline; CIP, ciprofloxacin; PEN, penicillin; AMP, ampicillin; GNH, high-level gentamicin; SXT, trimethoprim-sulfamethoxazole; N/D, not determined.

### MLST and molecular typing

**Figure 1:**
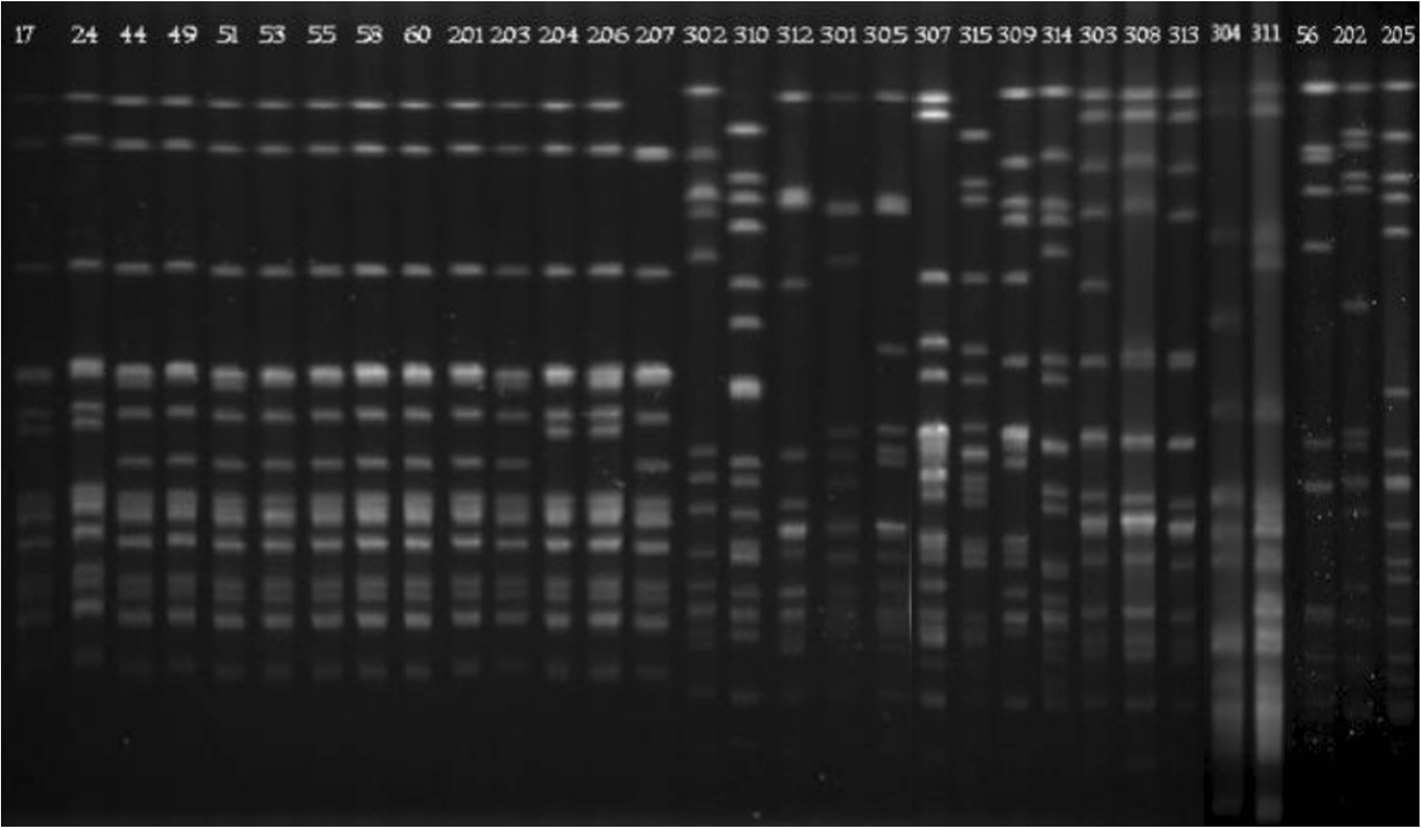
**PFGE of** ***Sma*** **I-digested chromosomal DNA of linezolid-resistant isolates.** Lane 17–207, fourteen isolates of LR *S. capitis*. 17, HS09-17; 24, HS10-24; 44, HS11-44; 49, HS12-49; 51, HS12-51; 53, HS12-53; 55, HS12-55; 58, HS12-58; 60, HS13-60; 201, HS12-201; 203, HS11-203; 204, HS10-204; 206, HS09-206; 207, HS13-207. Lane 302–311, fourteen isolates of LR *E. faecalis.* 302, HS13-302; 310, HS12-310; 312, HS13-312; 301, HS13-301; 305, HS11-305; 311, HS10-311; 307, HS11-307; 315, RJ13-315; 309, HS12-309; 314, RJ13-314; 303, HS13-303; 308, HS12-308; 313, HS13-313; 304, HS11-304; 311, HS10-311. Lane 56–205, three isolates of LR *S. aureus.* 56, HS12-56; 202, HS11-202; 205, HS09-205.

All of the isolates, except for those from *S. capitis*, were typed by MLST. The STs of the three *S. aureus* isolates were ST5, ST88 and ST239. The 14 *E. faecalis* isolates were classified into eight STs: two isolates each belonged to ST4, ST16, ST69, and ST476, while three were ST480 and three others belonged to different STs. The *E. faecium* isolate belonged to ST650. PFGE analysis showed that nine *S. capitis* isolates (HS11-203, HS11-44, HS12-49, HS12-201, HS12-51, HS12-53, HS2-55, HS12-58, HS13-60) exhibited identical band pattern, and four other isolates also shared the same band pattern (HS09-206, HS09-17, HS10-204, HS10-24). HS13-207 showed a unique band pattern (Figure 1). In addition, *E. faecalis* isolates from the same ST also exhibited different band patterns.

### Resistance genes and mutations

A novel C2131T mutation and a previously reported G2603T mutation, both in domain V of the 23S rRNA gene, were identified in all *S. capitis* isolates (Table 2). All *S. capitis* isolates, except HS09-206, HS09-17, HS10-204, HS10-24 and HS13-207, were positive for *cfr*. Furthermore, *S. capitis* HS12-201 and HS12-55 had C316T (Arg106Cys) mutations in ribosomal protein L4, but no mutations were detected in ribosomal proteins L3 or L22 in any isolates. Among *S. aureus* isolates, only HS12-56 was positive for *cfr. S. aureus* HS11-202 had a C389G (Ala130Gly) mutation in ribosomal protein L3, but proteins L4 and L22 were wild-type. No mutation was identified in any of the studied genes in the *E. faecalis* and *E. faecium* isolates, nor was *cfr* detected.

### Gene dosage

**Figure 2:**
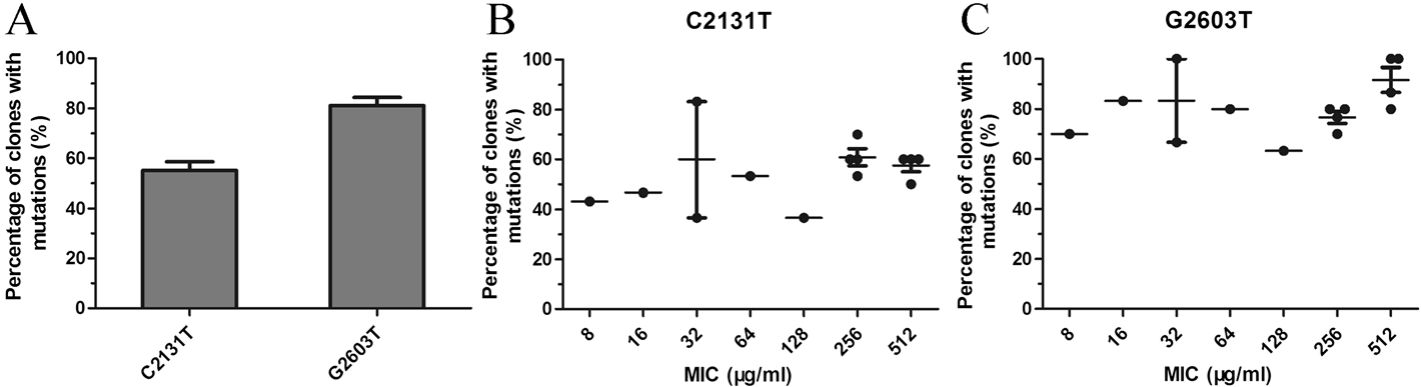
**Mutations in the 23S rRNA gene and linezolid resistance levels in LR** ***S. capitis*** **isolates. A**. The percentages of clones with C2131T and G2603T mutations. **B**. The percentage of clones with C2131T mutations in isolates with various linezolid resistance levels. **C**. The percentage of clones with G2603T mutations in isolates with various linezolid resistance levels. Error bars represent the mean ± SEM.

Gene dosage tests were performed on fourteen LR *S. capitis* isolates that contained mutations in domain V of the 23S rRNA gene. Isolates with different linezolid resistance levels displayed diverse C2131T and G2603T mutation rates (Table 2). The relationship between linezolid resistance levels and the percentage of mutated 23S rRNA genes in *S. capitis* is shown in Figure 2. Clones derived from *S. capitis* (linezolid MIC 8–64 μg/ml) had mutation rates of 36.7–83.3% (C2131T) and 63.3–100% (G2603T), and those derived from *S. capitis* (linezolid MIC 256–512 μg/ml) had mutation rates of 50.0–70.0% (C2131T) and 70.0–100% (G2603T).

### Genetic environment of cfr in plasmids

**Figure 3:**
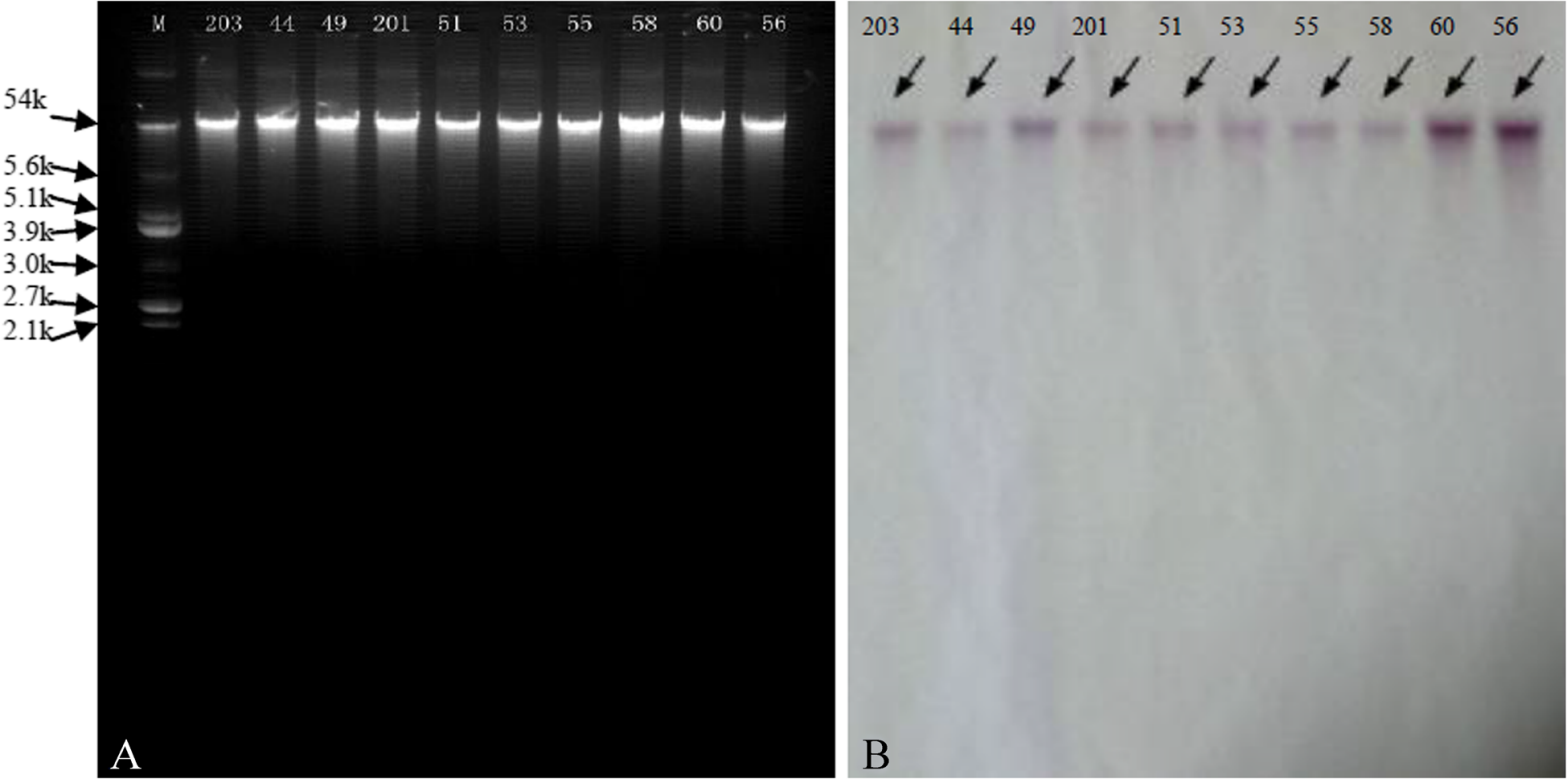
**Analysis of plasmids in** ***cfr*** **-positive** ***S. capitis*** **isolates. Plasmids of ca.54 kb were detected in all ten** ***cfr*** **-positive isolates. A**. Southern hybridisation of *S. capitis* isolates with a *cfr* probe. **B**. M, E. coli V517 marker; 203, HS11-203; 44, HS11-44; 49, HS12-49; 201, HS12-201; 51, HS12-51; 53, HS12-53; 55, HS12-55; 58, HS12-58; 60, HS13-60; 56, HS12-56.

**Figure 4:**
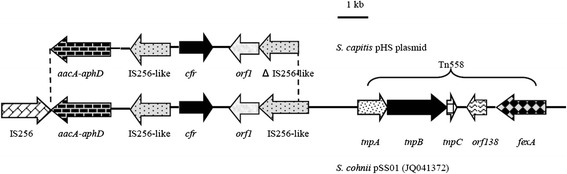
**Schematic representation of the genetic environment of** ***cfr*** **in pHS and pSS-01.** The arrows indicate the positions and directions of the transcription of the genes. The regions of homology between pHS and pSS-01 (GenBank accession no. JQ041372) are indicated by dashed lines and grey shading. Δ indicates a truncated gene.

Southern hybridisation indicated that *cfr* resided on plasmids of similar sizes (ca. 54 kb) in all ten *cfr-*positive isolates (Figure 3). The *cfr*-carrying plasmids were extracted from the isolates and transformed into *S. aureus* RN4220. Plasmids were then extracted from all the transformants and were confirmed by Southern hybridisation (data not shown). The linezolid MICs of ten transformants were 32 μg/ml. The genetic environment surrounding *cfr* in pHS (plasmid from Huashan Hospital) was shown to be identical in all ten *cfr*-carrying plasmids and showed 99% identity to the corresponding region of plasmid pSS-01 (GenBank accession no. JQ041372, Figure 4). The *cfr* gene was located downstream of aminoglycoside resistance gene *aacA-aphD* and was flanked by two copies of the IS256-like element, with a downstream *orf1* gene.

### Biofilm production

**Figure 5:**
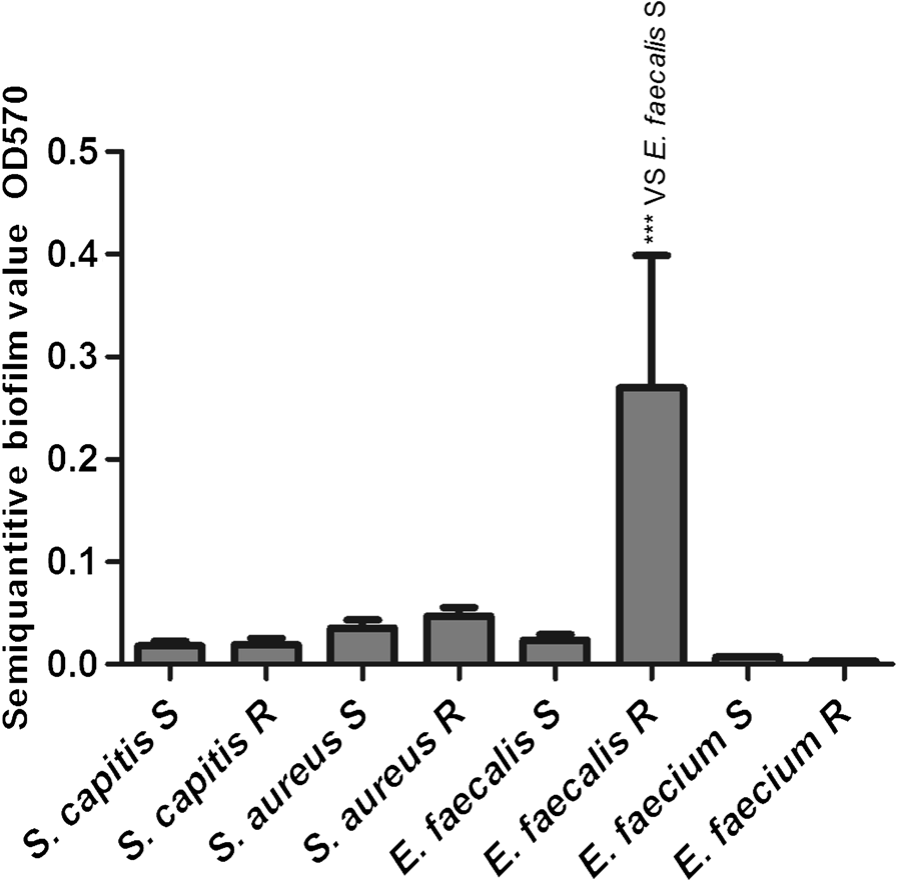
**Semi-quantitative biofilm analysis of** ***S. capitis, S. aureus, E. faecalis, E. faecium*** **groups.** LS strains were used as controls. Comparisons are vs. LS *E. faecalis* strains for LR *E. faecalis* strains. ***P < 0.0001 (one-way ANOVA).

The semi-quantitative biofilm assay showed that the mean biofilm production in LR *E. faecalis* strains was significantly higher than that in LS *E. faecalis* strains (*P* < 0.0001, one-way ANOVA), whereas there were no significant differences between LR and LS isolates from the other three species (Figure 5).

### Electron microscopy

**Figure 6:**
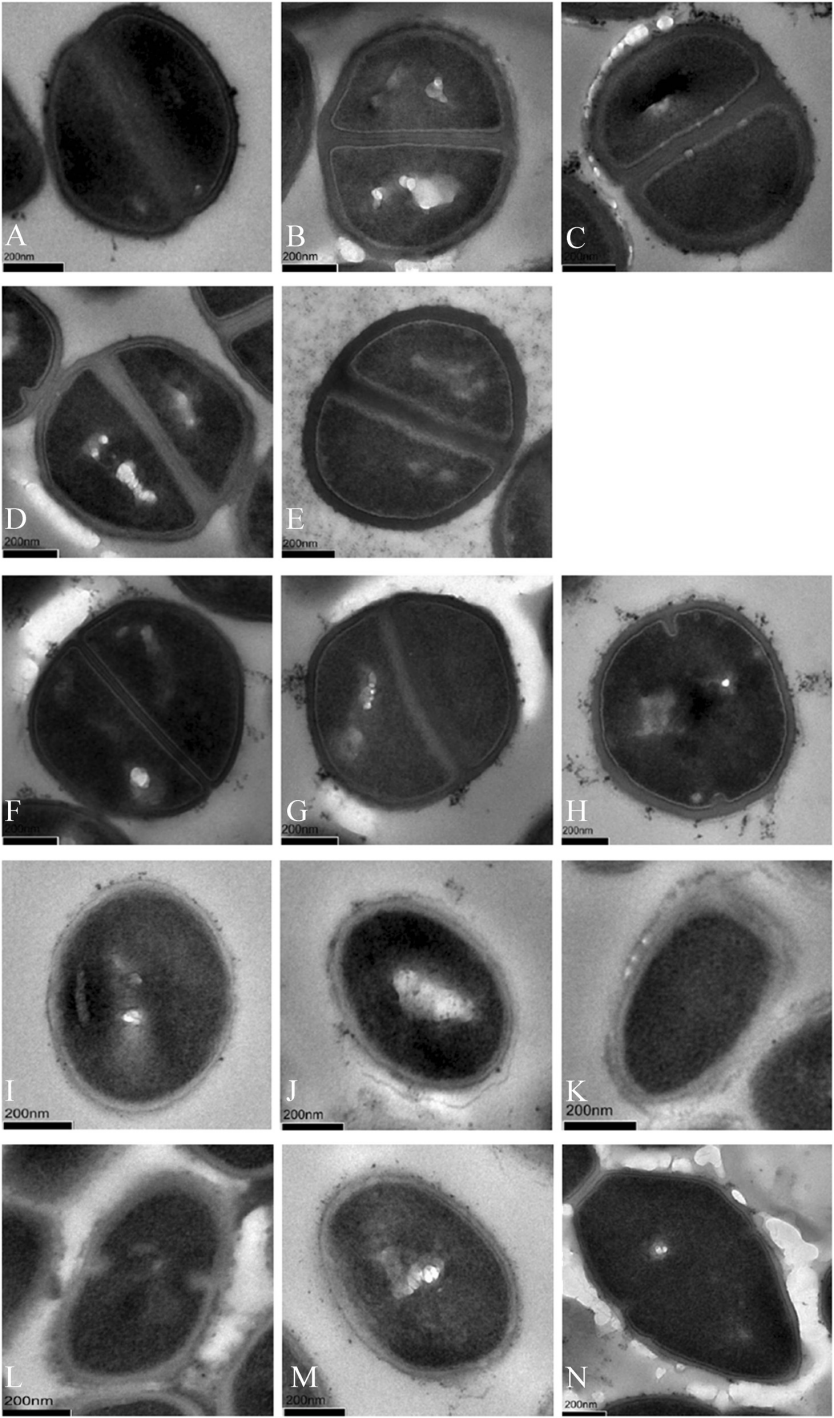
**Transmission electron micrographs (TEM) showing cell wall thickness in bacterial isolates.** TEM images show LS *S. capitis* HS12-102 **(A)**, LR *S. capitis* HS09-17 (MIC 16 μg/ml) grown without **(B)** and with **(C)** 4 μg/ml linezolid, LR *S. capitis* HS12-55 (MIC >256 μg/ml) grown without **(D)** and with **(E)** 32 μg/ml linezolid, wild-type ATCC29213 *S. aureus* **(F)**, LR *S. aureus* HS11-202 (MIC 16 μg/ml) grown without **(G)** and with **(H)** 2 μg/ml linezolid, wild-type ATCC29212 *E. faecalis* **(I)**, LI *E. faecalis* HS11-304 (MIC 4 μg/ml) grown without linezolid **(J)**, LR *E. faecalis* HS12-309 (MIC 8 μg/ml) grown without **(K)** and with **(L)** 2 μg/ml linezolid, LS *E. faecium* HS13-194 **(M)**, LI *E. faecium* HS11-306 (MIC 4 μg/ml) grown without linezolid **(N)**. Cell wall thicknesses are given in Table 3. Scale bars indicate 200 nm.

**Figure 7:**
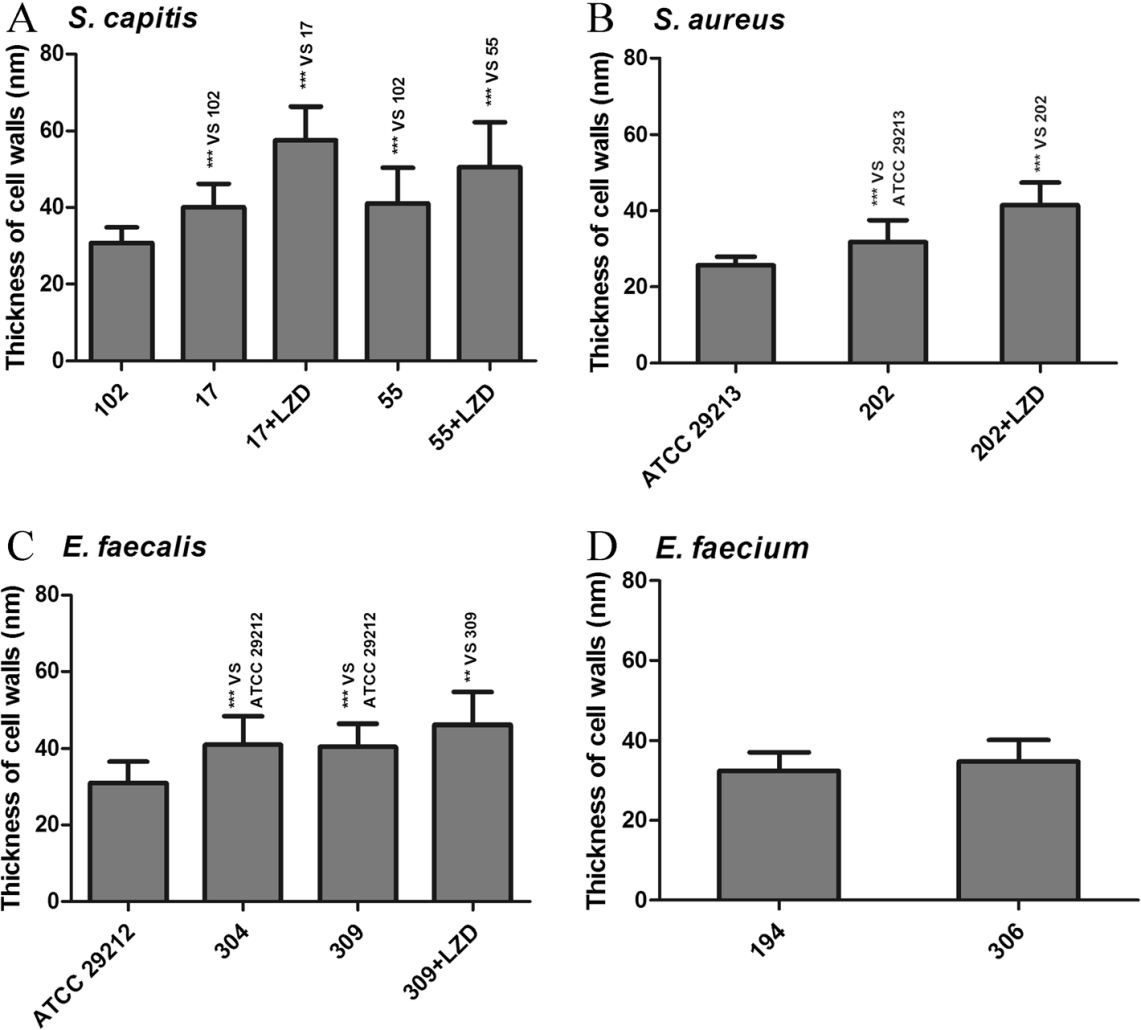
**Comparison between bacterial cell wall thickness in LS, LR and LI isolates.** There are *S. capitis* **(A)**, *S. aureus* **(B)**, *E. faecalis* **(C)** and *E. faecium* **(D)** isolates. 102, HS12-102; 17, HS09-17; 17 + LZD, HS09-17 grown with 4 μg/ml linezolid; 55, HS12-55; 55 + LZD, HS12-55 grown with 32 μg/ml linezolid; ATCC29213, ATCC29213 LS *S. aureus*; 202, HS11-202; 202 + LZD, HS11-202 grown with 2 μg/ml linezolid; ATCC29212, ATCC29212 LS *E. faecalis*; 304, HS11-304; 309, HS12-309; 309 + LZD, HS12-309 grown with 2 μg/ml linezolid; 194, HS13-194; 306, HS11-306 (MIC 4 μg/ml). LS reference strains HS12-102 (*S. capitis*), ATCC29213 (*S. aureus*), ATCC29212 (*E. faecalis*) and HS13-194 (*E. faecium*) were used as controls for the respective groups. 17 versus 102, 55 versus 102, 17 + LZD versus 17, 55 + LZD versus 55, 202 versus ATCC29213, 202 + LZD versus 202, 304 versus ATCC29212, 309 versus ATCC29212, 309 + LZD versus HS12-309 showed significantly differences (**P ≤ 0.001; ***P < 0.0001, two-tailed *t*-test).

TEM showed differences in cell wall thickness between LS, LI and LR strains grown with and without linezolid (Figure 6, Table 3). The overall cell diameter had not significant difference between any of the strains of each species (data not shown), but the cell walls of LI and LR strains were thicker than those of LS strains (*P* < 0.0001, two-tailed *t*-test),except for LI *E. faecium* HS11-306. Furthermore, the cell wall of LR strains grown with linezolid were thicker than that without linezolid (*P* < 0.0001, two-tailed *t*-test), except for LR *E. faecalis* HS12-309 (*P* ≤ 0.001, two-tailed *t*-test) (Figure 7).

**Table 3:** **Cell wall thickness measured by TEM**

**Species**	**Strains**	**Cell wall thickness** ^**a**^
*S. capitis*	HS12-102	30.76 ± 4.10
*S. capitis*	HS09-17	40.12 ± 6.04
*S. capitis*	HS09-17 + 4 μg/ml linezolid	57.54 ± 7.75
*S. capitis*	HS12-55	41.13 ± 6.22
*S. capitis*	HS12-55 + 32 μg/ml linezolid	50.52 ± 7.78
*S. aureus*	ATCC 29213	25.69 ± 2.17
*S. aureus*	HS11-202	31.74 ± 5.73
*S. aureus*	HS11-202 + 2 μg/ml linezolid	43.67 ± 6.30
*E. faecalis*	ATCC 29212	31.01 ± 5.57
*E. faecalis*	HS11-304	40.96 ± 7.41
*E. faecalis*	HS12-309	40.44 ± 5.38
*E. faecalis*	HS12-309 + 2 μg/ml linezolid	46.14 ± 7.55
*E. faecium*	HS13-194	32.42 ± 4.60
*E. faecium*	HS11-306	34.79 ± 5.38

^a^measurements (in nm) are means ± standard deviations from 30 cells per sample.

## Discussion

The prevalence of MRSA, VRS and VRE strains has presented a new challenge in antimicrobial medication. Linezolid is one of the few treatment options that is highly active against Gram-positive pathogens. However, LR strains have been increasingly reported in worldwide [2-7,9-14] and now have also emerged in China [8,17-19]. Although the incidence of linezolid resistance among Gram-positive organisms remains low, the emergence of LR strains is still of great concern.

Surveillance data in our study suggested that all the LR *S. capitis* were recovered from bacteraemia patients, whereas LR *S. aureus* (LRSA) came from respiratory tract specimens. Linezolid-resistant/intermediate enterococci were collected from blood, urine, wound, bile and prostatic fluid. All of the patients infected with LR staphylococci were critically ill, which is consistent with findings from previous studies [8,17-19]. A study in Spain [31] described that LR strains emerged after patients received three courses of linezolid for 600 mg every 12 h for 14 days. Interestingly, as shown in Table 1, only seven hospitalised patients (HS09-17, HS10-24, HS11-44, HS12-51, HS13-60, HS13-207 and HS11-307) in our study had received linezolid therapy (range 13–40 days) prior to the appearance of LR strains. The remaining patients, other than outpatients whose antibiotic drug histories were not determined, had not been exposed to linezolid. So, except for linezolid exposure, if there were other factors involved worth considering.

Nowadays, linezolid susceptibility of Gram-positive clinical isolates is mainly monitored through two surveillance programmes, the Zyvox Annual Appraisal of Potency and Spectrum (ZAAPS) Program in Europe and the Linezolid Experience and Accurate Determination of Resistance (LEADER) Program in USA. In 2014, the ZAAPS Program analysed the linezolid activity of 7972 g-positive clinical isolates collected over 9 years (2004–2012) from 73 medical centres in 33 countries on five continents [32]. Data showed that 0.11% of CoNS (8/6909 isolates), 0.02% of *S. aureus* (4/25148 isolates), 0.92% of *E. faecalis* (4/434 isolates) and 0.03% of *E. faecium* (1/333 isolates) isolates were resistant to linezolid. Gu et al. [33] summarized the linezolid susceptibility profile of staphylococci from the LEADER Program over 7 years (2004–2010). Data showed that 1.4% of CoNS (73/5202 isolates) and 0.05% of *S. aureus* (13/23077 isolates) isolates were resistant to linezolid. Our data showed that the incidence of LR strains in China was higher than that of the two surveillance programs, except for LRCoNS which was similar to that of the LEADER program. In particular, the incidence of LRCoNS in China was nine times higher than that of LRSA, and the incidence of LR *E. faecalis* was 15 times higher than that of LR *E. faecium*, suggesting that ongoing surveillance is necessary.

Recently, multifocal outbreaks of LR staphylococci have been reported, including *S. epidermidis* (France, Spain, Mexico, Italy, Brazil, China), *S. cohnii* (Mexico, China), *S. haemolyticus* (Mexico, Brazil, China), *S. sciuri* (China), *S. capitis* (Greece, China), *S. hominis* (Brazil), *S. lugdenesis* (Brazil) and *S. aureus* (Italy, Hong Kong, Brazil), and linezolid-resistant/intermediate enterococci, including *E. faecalis* (Poland, Ireland, China, Taiwan), *E. faecium* (Germany, Ireland, China) and *E. avium* (Poland) [8,18,19,32,34]. In our study, we detected multiple LR *S. capitis*, *S. aureu*s, *E. faecalis* and *E. faecium* isolates. Notably, the clinically-derived LRSA isolates characterised in our investigation are the first to be reported in China. Previous studies [8,18,19] described that most of LRCoNS in China exhibited high-level resistance (MIC 256 μg/ml), with very few low- and medium-level LR isolates, whereas in other countries [32,34], low to medium-level LRCoNS (8–32 μg/ml) were more common, with a lack of high-level LRCoNS strains. In contrast, the 14 LRCoNS isolates in our study exhibited a wide range of linezolid resistance levels, with MICs from 8 to 512 μg/ml. Three LRSA isolates exhibited linezolid resistance (MIC 8–32 μg/ml), which were higher than LRSA isolates from Italy, Hong Kong and Brazil (MIC 4–8 μg/ml). Thereinto, two of the LRSA isolates were methicillin-resistant and multidrug-resistant, whereas HS09-205 LRSA was methicillin-susceptible. Moreover, low-level resistance in linezolid-resistant/intermediate enterococci (MIC 4–8 μg/ml) was similar to previous studies [8,18,19,32,34]. MLST and PFGE results showed that LR *S. capitis,* LR *S. aureus, E. faecalis* and *E. faecium* were polyclonal, suggesting diverse STs were all vulnerable to linezolid exposure.

Known mechanisms of linezolid resistance in Gram-positive cocci include mutations in the 23S rRNA gene, acquisition of *cfr* and mutations of ribosomal proteins L3, L4 and L22. The novel mutation C2131T and the previously-described mutation G2603T in the V domain of the 23S rRNA were identified in all 14 LR *S. capitis* clones. Although G2603T was first reported in 2009 [9], this is the first report of this mutation in China. The novel mutation C2131T, whether it plays a role in linezolid resistance is uncertain and needs a further confirmation. But this revelation may provide new information for investigating the mechanism of linezolid resistance caused by mutations in the V domain of 23S rRNA gene. Because staphylococci possess five or six copies of the 23S rRNA gene, linezolid resistance caused by 23S rRNA mutations develop slowly. Besier et al. indicated that accumulation of single point mutations might be associated with gradually-increasing levels of resistance, in a mechanism known as “gene dosage” [28]. To gain insight into the relationship between gene dosage and 23S rRNA mutations, we examined the proportion of cloned PCR products with mutations. Our data showed that the percentage of clones with G2603T mutation was significantly higher than that with the C2131T mutation. Independent of other resistance mechanisms, various levels of the 23S rRNA mutations mediated low- to medium-level linezolid resistance (HS09-206, HS09-17, HS10-204, HS10-24, HS13-207; MICs: 8–64 μg/ml). Moreover, the synergistic action of mutations in the 23S rRNA gene and ribosomal proteins L3 and L4, as well as acquisition of *cfr* gene mediated high-level linezolid resistance (MICs: 128–512 μg/ml). However, the complicated background of resistance mechanisms involved and the biological characteristics of the clinical bacteria overlaid the relationship between gene dosage and linezolid resistance levels.

The *cfr* gene is often located on transferable plasmid and is thus considered to promote horizontal spread of linezolid resistance. In our study, 10 LR staphylococcal isolates (nine *S. capitis* and one *S. aureus*) harboured *cfr* gene and all the *S. aureus* RN4220 transformants showed that *cfr*-carrying pHS mediate medium-level linezolid resistance (MIC 32 μg/ml). Wang et al. had indicated the two copies of IS256-like elements played an important role in the dissemination of *cfr* in animal isolates [17]. Subsequently, similar structures were found in LR *S. capitis* from Zhejiang [18], LR *S. cohnii* from Beijing [19], and now LR *S. capitis* and LR *S. aureus* from Shanghai in the current study. Notably, the pHS with *cfr* flanked by IS256-like elements is the first to be reported in a LRSA strain (HS12-56) in this study. Moreover, compared with plasmid *S. capitis* MHZ (GenBank accession no. JX232067) from Zhejiang [18], *S. capitis* pHS possessed additional upstream aminoglycoside resistance gene *aacA-aphD*. Considering the first *cfr*-positive LR *S. capitis* strain was detected in 2011 and HS12-56 LRSA which carried the same plasmid emerged in 2012, it suggested that the pHS was most probably transferred from LRCoNS to LRSA. This finding presented significant concerns about the possibility of *cfr*-positive LRCoNS acting as reservoirs for linezolid resistance. Based on the information above, *cfr* flanked by two IS256-like elements may be transmitted from animal-derived isolates to human-derived isolates and spread horizontally within, or even between, bacterial species. Therefore, ongoing surveillance is essential to avoid the dissemination of linezolid resistance.

Mutations in ribosomal proteins L3, L4 and L22 of the peptidyltransferase centre also contribute to decreased susceptibility to linezolid [7,12-14]. In our study, mutation C316T (Arg106Cys) in L4 was first detected in two *S. capitis* clones (HS12-201, HS12-55) and mutation C389G (Ala130Gly) in L3 was first detected in *S. aureus* (HS11-202). None of the LR isolates contained mutations in L22. Because no other mutation was detected in *S. aureus* HS11-202, which has a linezolid MIC of 16 μg/ml, suggesting that the Ala130Gly mutation in L3 might be associated with low-level linezolid resistance.

Biofilm could provide a microenvironment for bacteria to survive. Bacteria in a biofilm aggregated into micelles which could withstand attacks by innate host defence mechanisms and evaded the threats of antibiotics [15]. We performed a semi-quantitative biofilm assay, which only showed that biofilm formation was significantly higher in LR *E. faecalis* isolates than in LS *E. faecalis* isolates. This suggested that formation of biofilm might increase low-level resistance to linezolid in LR *E. faecalis* (MICs 4–8 μg/ml).

We performed TEM to determine whether the decreased susceptibility of LI and/or LR strains was related to the thickness of the cell wall. We found that the cell walls of LI and LR strains were significantly thicker than LS strains. Also, the cell walls of LR strains grown with linezolid were thicker than those of LR strains grown without linezolid. These thicked cell wall bacteria were probably selected by the linezolid pressure and might be able to avoid clearance in patients undergoing antibiotic therapy. There was no significant differences in cell wall thickness between LI and LR strains. Data suggested that the thickness of the cell wall might decrease susceptibility to linezolid to a certain degree, but not play a major role in linezolid resistance. Furthermore, no significant differences in cell wall thickness were observed between LS HS13-194 and LI HS11-306 *E. faecium* isolates, however, we observed a thick transparent substance around the bacteria. Whether this substance might hinder linezolid absorption and result in resistance is unknown.

## Conclusion

In summary, various levels of LR strains have emerged in Shanghai, China in our study. Mutiple resistance mechanism were involved in these LR strains. Although the prevalence of resistance to linezolid remains low, the emergence of LR staphylococcal and enterococcal clinical isolates should prompt increased attention, especially for the horizontal dissemination of *cfr*, and surveillance of linezolid resistance in Gram-positive bacteria is of increasing importance.
